# Fatty Acid Binding Protein 3 (FABP3) and Apolipoprotein E4 (ApoE4) as Lipid Metabolism-Related Biomarkers of Alzheimer’s Disease

**DOI:** 10.3390/jcm10143009

**Published:** 2021-07-06

**Authors:** Maciej Dulewicz, Agnieszka Kulczyńska-Przybik, Agnieszka Słowik, Renata Borawska, Barbara Mroczko

**Affiliations:** 1 Department of Neurodegeneration Diagnostics, Medical University of Bialystok, 15-269 Bialystok, Poland; agnieszka.kulczynska-przybik@umb.edu.pl (A.K.-P.); renata.borawska@umb.edu.pl (R.B.); mroczko@umb.edu.pl (B.M.); 2 Department of Neurology, Jagiellonian University, 30-688 Kraków, Poland; slowik@neuro.cm-uj.krakow.pl; 3 Department of Biochemical Diagnostics, Medical University of Bialystok, 15-269 Bialystok, Poland

**Keywords:** FABP3, ApoE4, CSF biomarkers, lipids metabolism

## Abstract

Background: Lipid metabolism-related biomarkers gain increasing researchers interest in the field of neurodegenerative disorders. Mounting evidence have indicated the role of fatty acid-binding proteins and pathology lipid metabolism in Alzheimer’s Disease (AD). The imbalance of fatty acids (FA) and lipids may negatively affect brain functions related to neurodegenerative disorders. The ApoE4 and FABP3 proteins may reflect processes leading to neurodegeneration. This study aimed to evaluate the relationship between the CSF levels of FABP3 and ApoE4 proteins and cognitive decline as well as the diagnostic performance of these candidate biomarkers in AD and mild cognitive impairment (MCI). Methods: A total of 70 subjects, including patients with AD, MCI, and non-demented controls, were enrolled in the study. CSF concentrations of FABP3 and ApoE4 were measured using immunoassay technology. Results: Significantly higher CSF concentrations of FABP3 and ApoE4 were observed in AD patients compared to MCI subjects and individuals without cognitive impairment. Both proteins were inversely associated with Aβ42/40 ratio: ApoE4 (rho = −0.472, *p* < 0.001), and FABP3 (rho = −0.488, *p* < 0.001) in the whole study group, respectively. Additionally, FABP3 was negatively correlated with Mini-Mental State Examination score in the whole study cohort (rho = −0.585 *p* < 0.001). Conclusion: Presented results indicate the pivotal role of FABP3 and ApoE4 in AD pathology as lipid-related biomarkers, but studies on larger cohorts are needed.

## 1. Introduction

Alzheimer’s disease (AD) is a progressive, fatal and common neurodegenerative disease dependent on many pathological processes [1]. Genetics, demographic, lifestyle and metabolism factors contribute to the development of neuropathological changes. Accumulation of Amyloid β (Aβ) fibrils and insoluble plaques, neurofibrillary tangles (NFT) composed of hyper-phosphorylated Tau, neuronal and synaptic loss and atrophy of brain regions critical to memory are the most common characteristic features of AD [2]. This extensive and considerable neuropathology of AD is related to many mechanisms which are still not fully explained. The main characteristic of AD pathology is an extracellular accumulation of Aβ. One of the most toxic forms of Amyloid β seems to be Aβ1-42. The pathological form Aβ1-42 arises due to cleavage amyloid precursor protein (APP) by β/γ-secretases. This small form of amyloid aggregate and create Aβ senile plaques. Literature data suggest that the processes of Aβ1-42 and phospho tau (pTau181) production and generation of senile plaques and neurofibrillary tangles are more complicated than previously suspected and may be regulated by many different molecules, including proteins related to lipid metabolism [3,4]. Moreover, lipid-related molecules may be potential novel biomarkers reflecting different neuropathological mechanisms [5]. One of the widely studied aspects of AD are changes in lipid metabolism ongoing in the brain during the development of cognitive impairment [3,5,6]

The APOE ε4 allele is the strongest and most well-studied genetic risk factor for sporadic AD, and it is present in approximately 14% of the worldwide population [7,8]. Among the three isoforms of *APOE* gene (Apo-ε2, Apo-ε3, Apo-ε4) present in the general population, only the variant of ε4 has been identified as a genotype closely related to the risk of developing late AD [9]. In contrast, the APOE ε2 is the strongest genetic protective factor and is observed in about 8% of the population [7]. The estimated risk of developing AD for heterozygous *APOE*-ε4 allele increased three times and for homozygous 12 times compared to most common APOE ε3 carriers [9]. ApoE4 is a small 299 amino acid protein, one of the essential glycoproteins of amphiphilic apolipoproteins mainly expressed in hepatocytes, astrocytes, mono- and adipocytes [8]. In physiological conditions, ApoE is crucial for cholesterol transport, metabolism of lipids in the brain, neuronal growth, repair, and membranes remodelling [8,10]. However, it can also be involved in some pathological mechanisms ongoing in the brain, but the exact pathological pathway of ApoE has not been fully defined and understood. The presence of the *APOE*-ε4 is related to increased atrophy of crucial brains’ structures and cognitive impairment [11,12].

Moreover, it is suggested that the association of ApoE4 with amyloid pathology in the brain of patients with AD [8,12,13]. Some authors imply that ApoE is involved in the metabolism and clearance of Aβ [8,13,14,15]. Study Mouchard et al. revealed that ApoE fragments create complexes with Aβ, which results in reduced clearance and increased accumulation of amyloid β within the brain of patients with AD [13]. The concentration of ApoE has been assessed in CSF and plasma patients with AD [11,16,17,18]. However, the findings of quantifying studies have shown inconsistent results [11,17,19,20,21]. Furthermore, APOE may influence CSF ApoE levels [11,20]. The association of ApoE CSF levels with ApoE genotype and CSF Tau may suggest that it play a role in neurodegeneration [11].

Mounting evidence suggests that Fatty acid-binding protein 3 (FABP3), heart-type (hFABP), may influence neurodegeneration and probable AD development [22,23,24]. FABP3 is expressed in the heart and nervous system (e.g., cerebral neocortex and hippocampal CA1 and CA2 region), especially in dopaminergic, acetylocholinergic and glutamatergic neurones [25]. FABP3 play a pivotal role in membrane fluidity, neuronal synapse formation and intracellular lipids transport, especially arachidonic acid (ARA) [6,25,26]. Furthermore, FABP3 via ARA-mediated may indirectly influence aggregation of amyloid beta and alfa-synuclein (αSyn), leading to the formation of Aβ plaques [6,27,28]. Recent studies have shown elevated levels of FABP3 in the cerebrospinal fluid of AD patients compared to controls [23,29,30,31]. The association between elevated levels of FABP3 and atrophy of crucial brain structures in patients with pathological amyloid concentrations has been found [12,31]. Increased concentration of FABP3 is related to tau pathology and neurodegeneration [29,31]. Both ApoE4 and FABP3 appear to be essential proteins associated with lipid metabolism and neurodegeneration.

Still, relatively little is known about the potential diagnostic and therapeutic application of lipid metabolism-related proteins in patients with mild cognitive impairments (MCI) and AD. There are few literature data concerning concentrations of ApoE4 in CSF of AD patients and a lack of findings of the levels of this protein in CSF patients with MCI. Therefore, the present study aimed to measure the concentrations of ApoE4 and FABP3 in cerebrospinal fluid of patients with AD, MCI and non-demented subjects (CTRL) and compare them to classical biomarkers and a clinical score of cognitive impairment.

## 2. Materials and Methods

The study population involved *n* = 70 (*n* = 48 women, *n* = 24 men, 73 median years) subjects from the Department of Neurology, Jagiellonian University Hospital, Krakow, Poland, and included 34 AD patients, 18 subjects with MCI, and 18 non-demented controls. In the clinical diagnosis of study groups, standard medical examination, a physical and neurological examination, laboratory screening tests, a comprehensive neurocognitive evaluation and magnetic resonance imaging or computed tomography of the brain were used. Study population includes cases with sporadic Alzheimer’s disease. None of patients including in this research, testified that there was a history of Alzheimer’s disease in their family. Information on the past medical history of patients was also verified. Patients with alternations in CT or MRI, suggesting cerebrovascular disorder and subjects with increased albumin quotient (QAlb) indicating blood-CSF barrier dysfunction were excluded from the study. The diagnosis of AD and MCI were based on the recommendations from the National Institute on Aging and Alzheimer’s Association (NIA-AA) criteria [32,33]. Neuroimaging and neuropsychological examinations were combined with neurochemical findings (levels of Aβ1–42, Tau and pTau181, and values of the Aβ1–42/Aβ1–40 ratio) for the most accurate clinical diagnosis of AD and MCI patients. The Erlangen Score algorithm was used for the interpretation of CSF biomarkers [34]. Study participants were classified based on concentrations of classical AD biomarkers (Table 1). Dementia severity was assessed by MMSE score.

The control group consisted of people who did not have subjective memory disorders that did not fulfil the MCI criteria or recurrent headaches. A careful examination of subjects in the control group, with detailed analyses of the CSF, allowed for the exclusion of the symptoms’ organic background. No one of the control group subjects showed any significant alternations in the established biomarkers for AD (levels of Aβ1–42, Tau and pTau181). These findings were confirmed by the Erlangen Score of 0 points in all 18 subjects of this group.

### 2.1. Biochemical Measurements

Samples of CSF were obtained into polypropylene tubes by a lumbar puncture at the L4/L5 or L3/L4 interspace. All CSF samples were centrifuged, aliquoted and frozen −80 °C until analysis. Biochemical measurements of tested proteins (FABP-3 and ApoE4) in CSF and AD biomarkers (Aβ1–42, Aβ1–40, Tau, and pTau181) in CSF were performed in the Department of Neurodegeneration Diagnostics, Medical University of Bialystok, Poland. The concentrations of FABP3 and APOE were assessed with commercially available quantitative bead-based immunoassay (MILLIPLEX MAP Human Neuroscience Magnetic Bead Panel Merck KGaA, Darmstadt, Germany). The assay was performed following the manufacturer’s instructions, and samples were diluted 1:10. Washing steps were done using Biotek 405LS. For readout, the 96 well plates, a Luminex^®^ 200™ analyser (Luminex Corporation, Austin, TX, USA) were used. Standards and samples were run in duplicates with a coefficient of variance (CV) < 20%.

The concentrations of neurochemical dementia diagnostics (NDD) biomarkers were measured in CSF using IBL kits (RE59661, RE59651, Hamburg, Germany) for Aβ1–42, Aβ1–40 and Fujirebio kits (81572, 81574, Gent, Belgium) for Tau and pTau181 proteins.

### 2.2. Statistical Analysis

Statistical analysis was performed by nonparametric tests and analysis using e.g., the *PMCMRplus* package in the statistical software (RStudio Version 1.4.1106, Boston, MA, USA). The Shapiro-Wilk test revealed that the concentrations of the tested proteins did not follow a normal distribution. The comparison between AD, MCI, and the control group was performed using the Kruskal-Wallis test. Subsequently, significant differences between the levels of the tested groups were analyzed using the post hoc Dwass Steele-Critchlow-Fligner test to verify in which groups the difference was statistically significant. The results are presented as medians and interquartile ranges. Statistical significance was set at *p* < 0.05. Additionally, the receiver operating characteristic (ROC) curve and area under curve (AUC) analysis was used to determine the diagnostic usefulness of tested proteins as potential lipid-related biomarkers for AD.

## 3. Results

### 3.1. Concentrations of Potential Lipid-Related Proteins as Biomarkers Candidates

**Figure 1:**
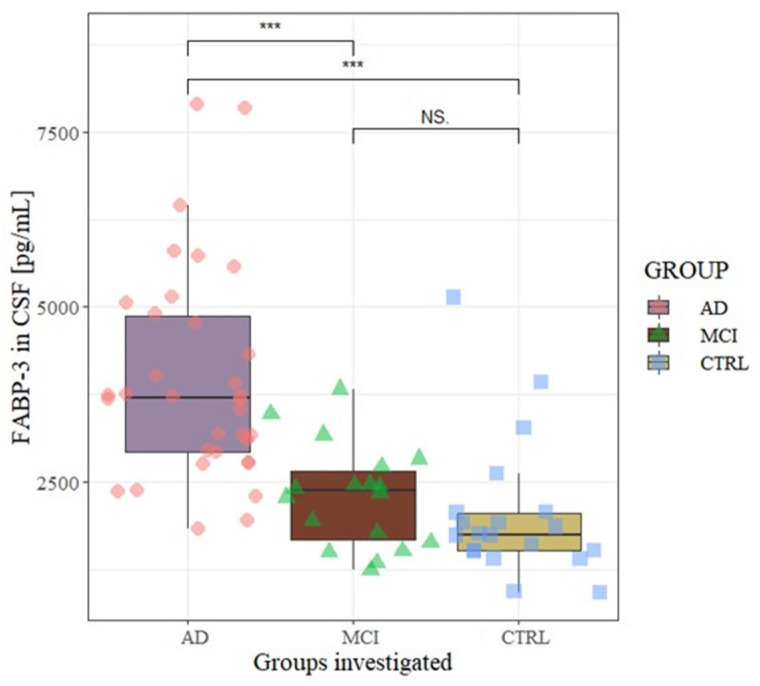
CSF levels of FABP3 in the analyzed groups. Level of statistically significant *** *p* < 0.001, NS—no significant. AD—Alzheimer’s Disease, MCI—mild cognitive impairment, CTRL—control, CSF—Cerebrospinal fluid.

The demographic and biochemical characteristics of study participants were presented in Table 1 and Table 2. Moreover, the concentrations of FABP3 and ApoE4 in the cerebrospinal fluid were presented (Table 1). Based on the Kruskal-Wallis test, the significant differences between all tested groups were observed for CSF levels of Aβ42/40 ratio (*p* < 0.001), Aβ42 (*p* < 0.001), Tau (*p* < 0.001), pTau181 (*p* < 0.001), ApoE4 (*p* < 0.001), FABP3 (*p* < 0.001). These differences were verified by the post hoc Dwass-Steele-Critchlow-Fligner test. The highest CSF concentration of FABP3 was observed in a group of patients with AD in comparison to MCI (*p* < 0.001) and controls (*p* < 0.001). In MCI patients, the CSF level of FABP3 was also higher than controls, but the difference was not statistically significant (*p* = 0.362) (Table 1, Figure 1).

A significantly higher concentration of ApoE4 was found in AD patients compared to MCI subjects (*p* = 0.003) and CTRL group (*p* = 0.009). In the MCI group, the CSF level of ApoE4 decreased compared to CTRL but not statistically significant (*p* = 0.08).

### 3.2. Associations between CSF Levels of FABP3, ApoE4 and Neurochemical Dementia Biomarkers (Aβ42/40 Ratio, Tau, pTau181)

**Figure 2:**
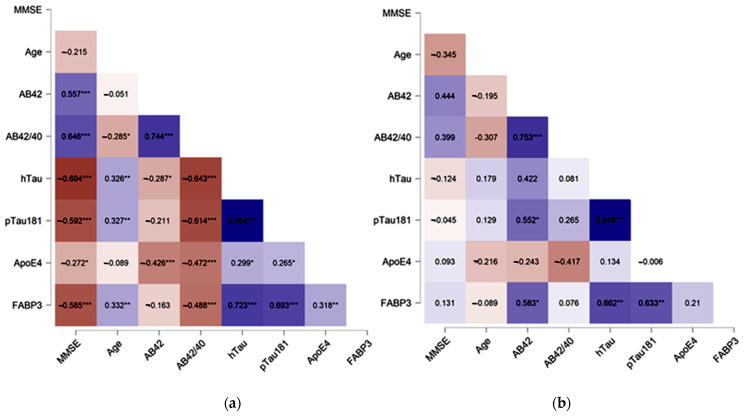
Spearman’s correlations between CSF tested proteins and neurochemical dementia biomarkers in the whole study group (**a**) and MCI subjects (**b**). NOTE Levels of statistical significant * *p* < 0.05, ** *p* < 0.01, *** *p* <0.001. MMSE—mini mental state examination score.

The associations between levels of FABP3, ApoE4 and neurochemical biomarkers were performed using the Spearman rank correlation test. In the whole study group (AD + MCI + CTRL) significant positive correlations between CSF levels of FABP3 and age (rho = 0.332, *p* = 0.002), Tau (rho = 0.723, *p* < 0.001), pTau181 (rho = 0.693, *p* < 0.001) and negative with: MMSE (rho = −0.585, *p* < 0.001), Aβ42/40 ratio (rho = −0.488, *p* < 0.001), ApoE4 (rho = 0.318, *p* = 0.007) (Figure 2a) were observed. In the same study group the levels of ApoE4 positively correlated with Tau (rho = 0.299, *p* = 0.012), pTau181 (rho = 0.265, *p* = 0.026) and negatively with MMSE (rho = −0.272, *p* = 0.023), Aβ42 (rho = −0.426, *p* < 0.001), as well as Aβ42/40 ratio (rho = −0.472, *p* < 0.001) (Figure 2a). Not observed associations between levels of FABP3 and ApoE4 in any AD and MCI compared group.

In contrast, in AD group the weak correlation was observed between FABP3 and Aβ42 (rho = 0.42 *p* = 0.04) and Tau (rho = 0.38, *p* = 0.03) as well as between ApoE4 and MMSE (rho = 0.34, *p* = 0.04).

In MCI group, CSF levels of FABP3 significantly correlated with the concentrations of Aβ42 (rho = 0.58, *p* = 0.03), Tau (rho = 0.66, *p* = 0.004) and pTau181 (rho = 0.63, *p* = 0.006).

In the group of non-demented controls was observed significant moderately strong correlation between FABP3 and ApoE4 (rho = 0.63, *p* < 0.01), and strong correlations with pTau181 (rho = 0.84, *p* < 0.001), as well as Tau (rho = 0.84, *p* = 0.001). In the same group ApoE4 significantly correlated with AB42/40 ratio (rho = 0.49, *p* = 0.04) and Tau (rho = 0.49, *p* = 0.04).

### 3.3. Diagnostic Usefulness of Candidate Biomarkers

**Figure 3:**
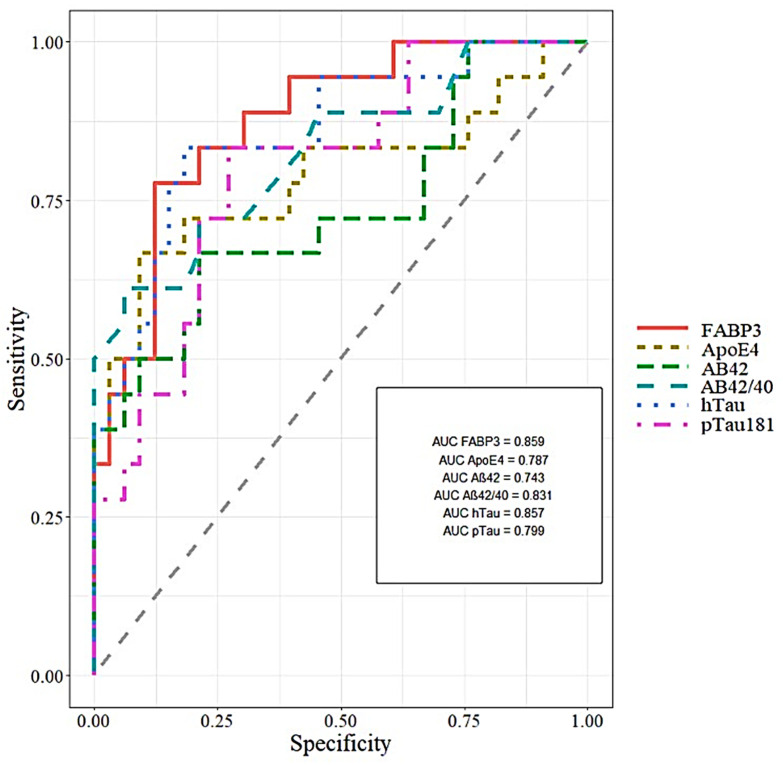
Areas under ROC curves (AUC) for CSF FABP3, ApoE4 and classical biomarkers in MCI compared to AD.

All tested proteins and classical biomarkers with the area under the curve (AUC) were presented in Table 3. The significant results of the receiver operating characteristic curve (ROC) were presented in Figure 3. The analysis of ROC was performed in MCI patients compared to AD. The AUC of FABP3 was slightly higher in comparison to classical biomarkers in MCI compared to the AD group. In the same group, the AUC of ApoE4 was slightly lower in comparison to classical biomarkers.

Analysis of ROC showed that CSF levels of FABP3 may significantly discriminate AD patients from controls (AUC = 0.881, *p* < 0.001), with 84.6% of accuracy, 88.2% specificity and 77.8% sensitivity. The ApoE4 concentration in CSF may significantly differentiate AD patients from controls (AUC = 0.751, *p* = 0.001), with 68% of accuracy, 80% specificity and 61.8% sensitivity.

## 4. Discussion

A non-negligible role in developing AD pathology and cognitive impairment has been attributed to disturbed homeostasis and metabolism of lipids, including fatty acid [5,35,36]. To our best knowledge, this is the first study analyzing the combination of CSF concentrations of lipid metabolism-related biomarkers, such as FABP3 and ApoE4 proteins with CSF levels of neurochemical dementia biomarkers (NDD).

Our study has shown significantly increased FABP3 concentrations in CSF of AD patients compared to MCI subjects and older people without cognitive decline. These findings are similar to previous studies, where the CSF levels of FABP3 in AD patients was also higher than in controls [23,29,31]. Contrary to our results are studies Guo et al., which demonstrated significantly higher levels of FABP3 in progressive MCI than cognitively healthy controls, but no difference between the AD dementia group and the progressive MCI sub-group [37]. Our results may suggest that CSF concentrations of FABP3 are already increased in the early clinical stages of AD and increased with the severity of the disease. The detectability of both proteins, such as FABP3 and ApoE4 in CSF of MCI patients may depend on many factors. Probably both tested proteins can be detected in the later stages of the disease due to the gradual potentiating disturbance of lipid metabolism. On the one hand, the pathological levels were closely related to changes in lipids metabolism, transport, and accumulation in crucial brain regions, like the hippocampus. On the other hand, in many cases, the pathological concentrations of these proteins, may also be associated with neurodegeneration and death of neurons releasing these molecules. However, it seems that the concentrations of these proteins are detectable only later, in an advanced stage of the disease, when the processes of destruction in CNS are extensive. We observed dynamics of changes in FABP3 and ApoE4 concentrations already in the early stages of the disease, although the differences are not statistically significant, which may indicate that they are rather later indicators of disease. It is worth noting, that a valuable feature of the biomarker is not only detecting in the early stages of the disease but also useful in differentiation with other neurodegenerative diseases, which could be pivotal in the case of FABP3 protein. Therefore they probably could be used for the prediction of clinical progression from MCI to AD.

Furthermore, the advantage of this protein in AD is the possibility to improve the differential diagnosis. The studies have demonstrated the highest concentrations of FABP3 in AD patients in comparison to other neurodegenerative disorders, such as Creutzfeldt–Jakob Disease (CJD), Parkinson’s Disease (PD) or Dementia with Levy Body(DLB) [29,31,37,38]. Three papers describing FABP3 levels in serum, but only one was related to Alzheimer’s disease and the other two of them to dementia with Lewy bodies (DLB) and proteomic studies performed on MCI Down Syndrome (DS) and also on AD-DS patients [39,40,41]. The highest levels of FABP3 were observed in patients with DLB and PD [39], what may indicate on the possibility of use it in differential diagnosis. Considering that FABP3 expression in the brain gradually increases in the grey matter after birth but lowers in the adult brain, which is crucial for developing axons, neurite formation, and maturation synapses [25,27]. We can suspect that increase the CSF concentration in AD patients might be a part of the disruption of lipids and fatty acids conditions ongoing in the brain [24,26,27,42,43]. The highest concentration of that protein in the AD patients and correlation with Tau and pTau181 in MCI subjects and Tau in AD patients may suggest the association with the neurodegeneration process. The fact that FABP3 in the brain may also regulate the neuronal membrane’s lipid composition could affect synaptic plasticity and cholinergic activity, glutamatergic, and especially GABAergic inhibitory interneurons [22,27,42,43]. We suggest that FABP3 play a pivotal role in the development of cognitive decline. FABP3 may also regulate dopamine D2R function in the striatum and anterior cingulate cortex (ACC), a crucial brain region of GABAergic interneurons responsible for coordinating cognitive processes [28,42,44]. FABP3 regulates GABA synthesis by transcriptional regulation of Gad67, which affects abnormal cognitive function and emotional behavior [42]. The downregulation of Gad67 in 5xFAD brains significantly reduced the Aβ plaques, one of the leading cause of developing AD and classical biomarker [42,45]. Moreover, in a similar way like FABP3 in GABAergic interneurons acts phosphorylated Tau protein, pTau primarily accumulated in GAD67 GABAergic interneurons, reduced GABAergic transmission CA1 mice brain and led to neuronal dysfunction [46].

The ApoE4 concentration is higher in AD patients in comparison to MCI and CTRL. However, MCI patients had lower and not significant levels of ApoE4 in CSF compared to non-demented controls. According to our best knowledge, immunoassay findings concerning the lack of the concentration of ApoE4 in CSF patients with dementia disorders. Most of the studies have demonstrated the blood and CSF levels of total ApoE in patients with different APOE alleles [16,20,21,47]. However, the results are inconsistent. The sensitivity and specificity of immuno- and biochemical assays depend on preanalytical and other factors, such as used type of antibodies, the platform for reading and quantifying the results, standards as well as controls. The specificity of immunoassays was controlled by precisely targeted antibodies to FABP3 and ApoE4, the manufacturer range of controls for the kit, and analysis of CV replicates. In addition, the manufacturer assured that there are no interactions between proteins, which could affect the specificity.

Some studies indicate increased levels of total ApoE [19,48,49]. Only a few papers have presented results of total ApoE concentration in the blood [19,21,50]. No one of searched papers was about measurement the concentrations of ApoE4 in blood by immunoassays methods. In one article researchers have been shown the levels of ApoE and their different isoforms in the plasma of AD patients and controls [21]. The authors of this paper conclude that, the ApoE plasma concentration were significantly decreased in APOE e4 carriers, which may be attributed to a specific ApoE4 isoform [16,21]. Others have provided data concerning a decreased or even unchanged levels of ApoE in AD compared to controls and concluded that the plasma ApoE concentration had no clinical significance [21]. Studies by Minta et al. have reported that among the three isoforms of ApoE in heterozygotes, the highest concentration was observed for ApoE4 (E2 < E3 < E3), which can be related to isoform-specific differences in Aβ clearance [11]. The highest CSF concentration of ApoE4 in AD patients included in our study can be connected to the accelerated accumulation of Aβ oligomers. In the brains of AD patients, the apoE4, after specific fragmentation, may bind to Aβ and slow down the clearance and favours deposition of the amyloid [8,9]. In vivo studies on APOE-ε4 mice have shown that clearance of Aβ was ineffective compared to mice with APOE-ε3 [10,14,51]. ApoE4 in the brain is lipidated by ATP-binding cassette transporters A1 (ABACA1) and G1 (ABCG1) and internalized in ApoE receptors such as low-density lipoprotein receptor-related protein 1 (LRP1). The LRP1, very-low-density lipoprotein receptor (VLDLR) and Apolipoprotein E receptor 2 (ApoER2) are also Aβ receptors [8]. The major pathway of Aβ clearance and take-up is closely related to receptor-mediated clearance (LRP1, LDLR) by neurons and glial cells in the brain parenchyma and vascular smooth muscle [8,10]. It is suggested that APOE might reduce Aβ deposition via knock-out the APOE gene or increasing the expression ABCA1 [52], which decreases Aβ deposition and plaques formation. Moreover, insufficient clearance may also be related to the glymphatic system and inadequate functioning and disruption of the blood-brain barrier [53,54]. The growing body of evidence suggests that ApoE is related to synaptic plasticity and destabilization of microtubules [5,14,55,56,57]. Disturbed clearance of Aβ also influences accumulation in the synaptic cleft, which disrupts synaptic transmission and long-term potentiation (LTP), one of the major processes related to memory and learning [56]. The dendritic spine density and length was reduced and hippocampal LTP was negatively altered in APOE-ε4 mice [56,58]. The pathological changes in reduced dendritic spine density and synaptic loss were also observed in AD patients brain tissues with APOE-ε4 [48]. Based on the available literature data, it can be hypothesized that ApoE4 impacts the molecular pathology of AD through impairment of astrocyte, microglia and Aβ clearance [59]. Moreover, ApoE4 influence abnormalities of lipid metabolism in astrocytes and microglia [59,60]. The isogenic human *APOE4* astrocytes contained more unsaturated triacylglycerides and accumulated lipid droplets (LDs) [60]. These pathological state of lipidome may be restored to the basal state by supplementing choline to the culture medium [60]. This research sheds new light on a potential pathway of influence and the importance of *APOE4* in AD pathology and could also be the starting point for drug research. The LDs store lipids and fatty acids in the cytoplasm as energy-rich reservoirs and fatty acids inside the cells [61]. Fatty acids into the cell are delivered, among others, by FABP proteins, including FABP3. ApoE4 disrupts neuronal functions by decreasing FA sequestering in lipid droplets [62]. Additionally, ApoE4 negatively modulated the internalization of LD, their transport to astrocytes and lower FAs oxidation [62]. Impaired transport and oxidation lead to lipids accumulation in the astrocytes and hippocampus [62]. Consequently, FA homeostasis is disrupted, leading to energy deficits, lipid dysregulation, and increasing AD risk in ApoE4 carriers [62]. Our research showed the moderate negative correlation of ApoE4 with Aβ42 and Aβ42/40) in the whole study group and positive with MMSE in the AD group. These results may underline an association the ineffective Aβ clearance, which may lead to the creation of amyloid plaques and the development of cognitive decline. Moreover, not strong but significant correlations between ApoE4 and Tau as well as pTau181 were found what is in line with previous studies [11]. They may indicate the possible association of ApoE4 with degeneration of neurons. However, still little is known about that dependency. In the present study, CSF levels of FABP3 were strongly associated with Tau and pTau181 in the whole study group, MCI subjects, and Tau protein in AD patients.

Additionally, a negative correlation between CSF FABP3 levels and MMSE score was found in the whole study group, similar to other reports [29]. These findings confirm that change in the CSF levels of FABP3 may reflect lipid-related mechanism in the course of ongoing neurodegenerative processes and cognitive impairment. Hence, FABP3 seems to be useful as a potential biomarker of neuronal degeneration. Changes in the concentrations between the tested proteins and classical biomarkers, may be a consequence of increasing pathological processes. Both of these proteins play important physiological roles in the healthy central nervous system, but the pathological levels may be depend on other factors. A many processes and interactions between proteins are still undiscovered, which is an excellent field for further research. Additionally, we revealed the association of FABP3 with ApoE4 in the whole cohort. In agreement with our findings is a study by Desikan et al., which showed a significant association between FABP3, ApoE and Aβ as an essential modifier of neurodegeneration and amyloid deposition [12]. These findings suggest an important relationship between neuronal lipids and neurodegeneration closely related to amyloid pathology and brain atrophy [12]. Considering the above studies, it can be speculated that FABP3, ApoE4 and Aβ have synergistic effects on AD pathology.

We assessed the diagnostic usefulness of tested proteins based on AUC results. The AUC results of FABP3 were comparable with classical biomarkers in the MCI group compared to AD patients. However, differentiation between AD and CTRL has shown less discriminatory capability than classical biomarkers. These results are consistent with the findings of metaanalysis of Olsson et al. [63]. Where researchers reported the moderate effect sizes and utility in differentiating AD from CTRL [63]. Studies of other researchers also demonstrated the high AUC for FABP3 in differentiating between AD and CTRL, but lower than classical biomarkers [31]. However, Chiaserrini et al. reported that combined AUC of two biomarkers the FABP3 and Tau increased the accuracy of differential diagnosis in the dementia group (AD vs. Dementia with Levy body (DLB)) [31]. The AUC values for ApoE4 were lower than classical biomarkers or FABP3 in both comparison group of patients. Despite of the fact that, our results of ApoE4 in CSF allowed us to differentiate AD from CTRL and AD from MCI patients, we are not able to confirm it the clinical utility of the protein. The opinions on the usefulness of assessing the ApoE4 as a biomarker in AD diagnostics are controversial [11]. Additionally, was performed an analysis of ROC and AUC with FABP3 and ApoE4 together, but the results did not improve the discriminatory ability between the all compared groups. Based on the available literature data, it can be hypothesized that ApoE4 impacts the molecular pathology of AD through impairment of astrocyte, microglia and Aβ clearance [59]. Moreover, ApoE4 influence abnormalities of lipid metabolism in astrocytes and microglia [59,60]. Lipids levels alter with ageing and may also be manipulated by diet, supplementation or gut microbiome [64]. The isogenic human *APOE4* astrocytes contained more unsaturated triacylglycerides and accumulated lipid droplets (LDs) [60]. These pathological state of lipidome may be restored to the basal state by supplementing choline to the culture medium [60]. This research sheds new light on a potential pathway of influence and the importance of *APOE4* in AD pathology and could also be the starting point for drug research. The LDs store lipids and fatty acids in the cytoplasm as energy-rich reservoirs and fatty acids inside the cells [61]. Fatty acids into the cell are delivered, among others, by FABP proteins, including FABP3. ApoE4 disrupts neuronal functions by decreasing FA sequestering in lipid droplets [62]. Additionally, ApoE4 negatively modulated the internalization of LD, their transport to astrocytes and lower FAs oxidation [62]. Impaired transport and oxidation lead to lipids accumulation in the astrocytes and hippocampus [62]. As a consequence, FA homeostasis is disrupted, leading to energy deficits, lipid dysregulation, and increasing AD risk in ApoE4 carriers [3,27,42,45,62,65,66].

Lipids studies point to additional aspect of ApoE4 in AD pathology [5,13,56,59,62,67]. Dysregulation of lipids and their roles in neurodegenerative diseases is an essential topic for investigating a novel biomarkers to diagnose and predict disease progression. It is possible that FABP3 and ApoE4 might have a common metabolic pathway closely related to the regulation of fatty acid metabolism across neurons and astrocytes. However, further research are needed to support these suppositions. FABP3 is a more promising biomarker in differentiating AD from CTRL and MCI than ApoE4. Studies by other researchers show the usefulness of FABP3 in differential diagnosis, not only in AD. FABP3 and ApoE4 as candidates for lipid metabolism-related biomarkers appear promising, but their differential effect is rather moderate. To unequivocally demonstrate diagnostic utility, studies should be conducted at other research centres on larger groups of subjects.

## 5. Conclusions

In summary, the presented research demonstrated that FABP3 and ApoE4 concentrations in CSF of AD patients are higher than those in MCI and older non-demented subjects. Moreover, the concentrations of FABP3 increased with the severity of the disease, hence it probably could be used to predict progression from MCI to AD. However, clinical utility of the measurement of CSF concentrations of ApoE4 protein seems to be limited. Further research on a larger cohort are needed. Our results further confirm and highlight the role of lipids and lipid-associated proteins in AD pathology. Research on the various lipid-related proteins could improve understating biological mechanisms underlying AD pathology.

## Figures and Tables

**Table 1:** The concentrations of tested proteins in the study groups.

Tested Variables	Median (Interquartile Range)			*p* (Kruskal-Wallis Test)	*p* (Dwass-Steele-Critchlow-Flinger Test)		
	AD	MCI	Controls		AD vs. CTRL	AD vs. MCI	MCI vs. CTRL
Aβ42/40 ratio CSF	0.03 (0.02–0.04)	0.05 (0.03–0.08)	0.07 (0.06–0.08)	<0.001	<0.001	<0.001	<0.001
Tau (pg/mL)	669 (561–943)	389 (327–495)	221 (190–256)	<0.001	<0.001	<0.001	<0.001
pTau181 (pg/mL)	83 (69–111)	57 (46–68)	38 (34–41)	<0.001	0.001	<0.001	0.002
ApoE4 (ng/mL) CSF	348,552 (8491–439,189)	4491 (3911–157,341)	9021 (6556–10,126)	<0.001	0.009	0.002	0.080
FABP3 (pg/mL) CSF	3704 (2937–4872)	2380 (1669–2651)	1752 (1514–2061)	<0.001	<0.001	<0.001	0.362

**Table 2:** Demographic data and characteristics of the study groups.

	Median (Interquartile Range)		
	AD *n* = 34	MCI *n* = 18	CTRL *n* = 18
Age (years)	76 (68–81)	75 (70–78)	68 (64–75)
Gender (Female/Male)	26/8	10/8	12/6
MMSE score (range 0–30 p.)	22 (19–24)	27 (26–29)	29 (27–30)

**Table 3:** AUC of tested parameters in compared groups.

Tested Parameters	ROC Criteria in AD Compared to CTRL				ROC Criteria in MCI Compared to AD			
	AUC	SE	95% C.I. (AUC)	*p* (AUC = 0.5)	AUC	SE	95% C.I. (AUC)	*p* (AUC = 0.5)
FABP3	0.881	0.046	0.7646–0.9968	<0.001	0.859	0.050	0.7569–0.962	<0.001
ApoE4	0.751	0.067	0.6195–0.8838	0.001	0.787	0.062	0.6349–0.9403	<0.001
Aβ42	0.930	0.034	0.8613–0.9998	<0.001	0.743	0.068	0.5909–0.896	<0.001
Aβ42/40	1	0	1	<0.001	0.831	0.055	0.7083–0.9551	<0.001
pTau181	0.969	0.022	0.9242–1	<0.001	0.799	0.060	0.6725–0.9255	<0.001
Tau	0.985	0.015	0.9614–1	<0.001	0.857	0.050	0.746–0.9696	<0.001

## Data Availability

The data presented in this study are available on request from the corresponding author. Key data are stated in the text.
